# A Rare Presentation Characterized by Epileptic Spasms in *ALDH7A1*, Pyridox(am)ine-5′-Phosphate Oxidase, and *PLPBP* Deficiency

**DOI:** 10.3389/fgene.2022.804461

**Published:** 2022-04-12

**Authors:** Xianru Jiao, Pan Gong, Yue Niu, Yuehua Zhang, Zhixian Yang

**Affiliations:** Department of Pediatrics, Peking University First Hospital, Beijing, China

**Keywords:** epileptic spasm, ALDH7A1, pyridoxine-dependent epilepsy, pyridox(am)ine-5′-phosphate oxidase deficiency, PLPBP

## Abstract

**Objective:** To analyze the clinical feature, treatment, and prognosis of epileptic spasms (ES) in vitamin B6–dependent epilepsy, including patients with pyridoxine-dependent epilepsy (PDE) caused by *ALDH7A1* mutation, pyridox(am)ine-5′-phosphate oxidase (*PNPO*) deficiency, and *PLPBP* deficiency.

**Methods:** We analyzed data from a cohort of 54 cases with PDE, 13 cases with *PNPO* deficiency, and 2 cases with *PLPBP* deficiency and looked for the presentation of ES among them.

**Results:** A total of 11 patients with the seizure presentation of ES have been collected. Among them, four patients carried mutations in *ALDH7A1*, six carried mutations in *PNPO*, and the remaining one carried mutation in *PLPBP*. The analysis of this cohort identified nine cases presenting as infantile spasms distributed in the three diseases and two cases presenting as Ohtahara syndrome diagnosed with PDE and *PNPO* deficiency, respectively. In the PDE and *PLPBP* deficiency groups, seizures were controlled by pyridoxine monotherapy, and the remaining one had refractory seizures due to secondary brain atrophy. In the groups with *PNPO* deficiency, one patient showed seizure-free when treated by PLP combined with valproic acid, three still had infrequent seizures treated by PLP monotherapy or pyridoxine or PLP combined with other antiseizure medications, and two died. In two cases presenting as Ohtahara syndrome, after regular treatment, one showed seizure-free, the others showed a marked decrease in seizure frequency, and they both showed an improvement in EEG.

**Significance:** ES might be a common form of seizures in *PNPO* deficiency, and EEG presented as hypsarrhythmia or a burst suppression pattern. It is difficult for pyridoxine to control frequent seizures caused by secondary brain injury. In our *PNPO* deficiency cohort, patients with infantile spasms did not respond better to PLP than pyridoxine. Timely and correct treatment could prevent the transformation of the child’s disease from Ohtahara syndrome and infantile spasms to subsequent epileptic encephalopathy or refractory epilepsy.

## Introduction

Vitamin B6–dependent epilepsy is a heterogeneous group of disorders characterized by early onset seizures which respond to pyridoxine or pyridoxal-5′-phosphate (PLP) due to decreased availability of the active cofactor PLP (Hunt et al., 1954; Mills et al., 2005; Mills et al., 2006; Darin et al., 2016). Pyridoxine-dependent epilepsy (PDE, OMIM: 266100) or *ALDH7A1* deficiency (traditionally) and pyridox(am)ine-5′-phosphate oxidase (*PNPO*) deficiency (OMIM: 610090) are the most prevalent type (Stockler et al., 2011). Both PDE and *PNPO* deficiency are recessively inherited disorders, caused by pathogenic variants in *ALDH7A1* and in *PNPO*, respectively (Mills et al., 2005; Mills et al., 2006). In 2016, biallelic pathogenic variants in *PLPBP* have been shown to cause a form of early onset vitamin B6–dependent epilepsy (OMIM: 610090) (Darin et al., 2016; Plecko et al., 2017). So far, more than 300 cases of PDE, 87 cases of *PNPO* deficiency, and 44 cases of *PLPBP* deficiency have been reported, respectively (Heath et al., 2020; Jiao et al., 2020; Alghamdi et al., 2021).

From a clinical point of view, literature data reported various types of seizures in patients with vitamin B6–dependent epilepsy, including focal seizures, generalized tonic-clonic seizures (GTCS), myoclonic seizures, and epileptic spasms (ES) (Yeghiazaryan et al., 2012; Falsaperla et al., 2018; Heath et al., 2020; Jiao et al., 2020; Alghamdi et al., 2021). In terms of ES, of the cases for which clinical data were available, 14% (27/188) of patients with PDE presented as ES (Basura et al., 2009; Bennett et al., 2009; Mills et al., 2010; Scharer et al., 2010; Yeghiazaryan et al., 2012; Pérez et al., 2013; Tlili et al., 2013; Mefford et al., 2015; van Karnebeek et al., 2016; Al Teneiji et al., 2017; Falsaperla et al., 2018; Jiao et al., 2020). In *PNPO* deficiency and *PLPBP* deficiency, the proportion was 10% (5/50) and 14% (5/36), respectively (Mills et al., 2014; Guerin et al., 2015; Olson et al., 2017; Xue et al., 2017; Heath et al., 2020; Mohanlal et al., 2020). To date, we have collected 54 cases of PDE, 13 cases of *PNPO* deficiency, and 2 cases of *PLPBP* deficiency. Here, we focus on the presentation of ES, a rare type of seizure in vitamin B6–dependent epilepsy, in our large cohort, including the three mutation types described above. At the same time, we analyzed the clinical characteristics, corresponding treatment, and prognosis of patients in each mutation type.

## Methods

### Ethics Statement

This study was approved by the Biomedical Research Ethical Committee of Peking University First Hospital. Written informed consent was obtained from the legal guardians (parents) of the patient for diagnostic procedures and next-generation sequencing (NGS).

### Patients

**FIGURE 1:**
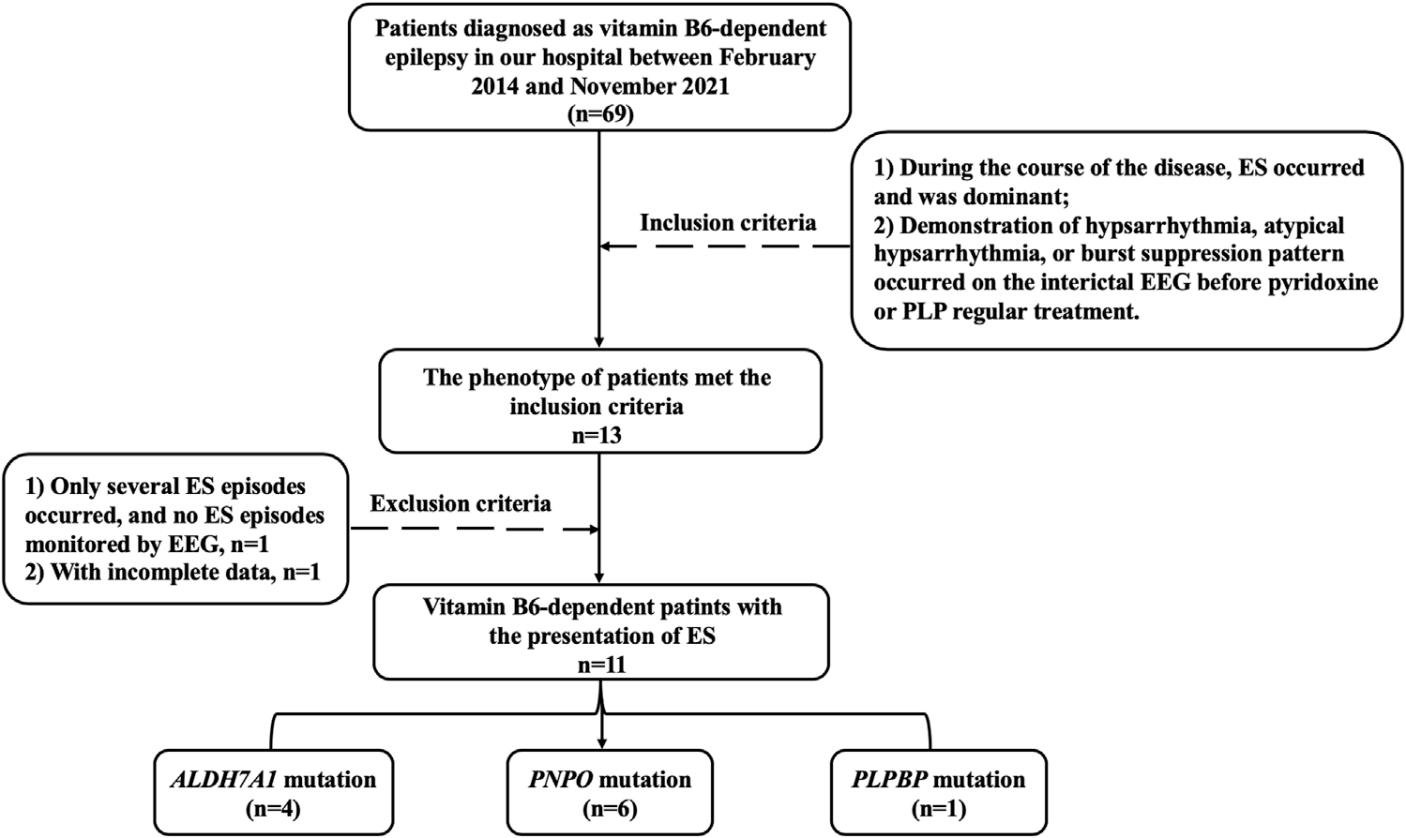
Data screening flow chart. ES, epileptic spasms; EEG, electroencephalogram; PLP, pyridoxal-5′-phosphate.

In general, there were 69 patients diagnosed with vitamin B6–dependent epilepsy in our hospital between February 2014 and November 2021. Based on that, we looked for cases with the presentation of ES. The following criteria were established: 1) during the course of the disease, ES, characterized by brief, symmetric axial muscle contraction (neck, trunk, and/or extremities), occurred and was dominant in the course of the disease and 2) demonstration of hypsarrhythmia, atypical hypsarrhythmia (“atypical” or “modified” hypsarrhythmia consists of asymmetric features, focal discharges, and semi-periodic burst-suppression), or burst suppression pattern occurred on the interictal electroencephalogram (EEG) before pyridoxine or PLP regular treatment. Records of patients who had not performed the adequate neurologic diagnostic chart were excluded from our analyses. We also excluded patients with only several ES episodes during the course of the disease and no isolated or series of ES episodes monitored by EEG (Figure 1).

The following data were collected from each patient: patient demographic data (including age and sex), anomalies during pregnancy, post-partum and perinatal anomalies, gestational age, clinical manifestations, treatment processes and outcomes, cognitive and behavioral development, EEG, brain magnetic resonance imaging (MRI), biochemical studies, and plasma amino acids and urine organic acids test. Neurodevelopmental assessment of these patients was evaluated through intelligence tests (Wechsler or Gesell intelligence scales). The intellectual disability (ID)/developmental delay (DD) severity was classified according to intelligence quotient (IQ) scores as mild (IQ of 55–70), moderate (IQ of 40–54), severe (IQ of 25–39), or profoundly severe (IQ of less than 25).

### Genetic Analysis

DNA extracted from peripheral blood from patients and other family members was analyzed using trio-based exome sequencing, which was performed at the local genetic institutes using NGS techniques according to standard procedures. Sequence variants were checked with population database gnomAD (http://gnomad.broadinstitute.org/) and the Combined Annotation-Dependent Depletion (CADD, https://cadd.gs.washington.edu/) score, and functional impact of mutations was predicted using three different *in silico* tools, including Polyphen2, SIFT, and Mutation Taster. Variant pathogenicity was interpreted according to the American College of Medical Genetics (ACMG) guidelines (Richards et al., 2015). The variants were further confirmed by Sanger sequencing. The deletion of exons was validated by quantitative polymerase chain reaction (qPCR).

## Results

A total of 11 patients presenting as ES have been described in our cohort, and nine have survived to date. Clinical characteristics and genetics are summarized in Table 1. Among them, four patients carried mutations in the *ALDH7A1*, six carried mutations in the *PNPO*, and the remaining one carried mutation in the *PLPBP*.

**TABLE 1:** Clinical characteristics and genetics of 11 patients with the presentation characterized by epileptic spasms.

	Current age/gender	GA	Birth history	Age at seizure onset	Age at the onset of ES	Seizure type	Age at which regular treatment started	Current medication	Interictal EEG/age	Brain MRI	Cognitive and behavioral development	Epileptic syndrome	Mutation information
*ALDH7A1* deficiency													
Patient 1	8y7m/F	At term	Normal	1m	7m	FS, ES	4y	PN	Hypsarrhythmia/2y	Normal	Moderate ID/GDD	IS	c.1547A > G (p.Y516C); c.1061A > G (p.Y354C)
Patient 2	4y6m/F	At term	Normal	4m	4m	ES	4m	PN	Hypsarrhythmia/4m	Normal	Severe ID/GDD	IS	c.563T > C (p.V188A); exon 1 deletion
Patient 3	3y5m/M	At term	Hypoxia	15d	15d	ES	35d	PN	Burst suppression/35d	CCH	Moderate ID/GDD	OS	c.1008+1G > A; exon 6 deletion
Patient 4	4y2m/M	At term	Hypoxia	1m	1m	ES, GTCS, FS, SE	3y	PN, VPA,LEV,LTG	Hypsarrhythmia/2y	Brain atrophy	Severe ID/GDD	IS	c.1547A > G (p.Y516C); c.1553G > C (p.R518T)
*PNPO* deficiency													
Patient 5^a^	11y/M	35^+5^w	Premature birth; hypoxia	<24 h	<24 h	ES,FS,GTCS,SE	2m	PN, PB	Atypical hypsarrhythmia/70d	PWMA, BVW	Severe ID/GDD	IS	c.445_448del; c.481C > T (p.R161C)
Patient 6^a^	Died at 8y/M	35^+5^w	Premature birth; hypoxia	<24 h	<24 h	ES,FS,GTCS,SE	2m	N.A.	Atypical hypsarrhythmia/70d	PWMA, BVW	Severe ID/GDD	IS	c.445_448del; c.481C > T (p.R161C)
Patient 7	4y10m/M	34^+1^w	Premature birth	<24 h	<24 h	ES, FS, MS	2m	PLP	Burst suppression/14d	Normal	Severe ID/GDD	OS	c.445_448del; c.344G > A (p.S115N)
Patient 8	Died at 1m/M	At term	Normal	<24 h	<24 h	ES,MS	N.A.	N.A.	Hypsarrhythmia/20d	N.A.	N.A.	IS	c.748_749del; c.482G > A (p.R161H)
Patient 9	4y/F	34^+5^w	Premature birth	<24 h	40d	FS,ES	8m	PLP,VPA	Hypsarrhythmia/3m	CCH	Severe ID/GDD	IS	c.445_448del; c.233C > A (p.A78D)
Patient 10	8y2m/M	At term	Normal	10d	70d	GTCS,ES,FS,SE	5y	PN,VPA,LTG	Hypsarrhythmia/3m	CCH	Severe ID/GDD	IS	c.445_448del; c.412C > T (p.R138C)
*PLPBP* deficiency													
Patient 11	5y/F	At term	Normal	7d	3m	FS,ES,SE	3m	PN	Hypsarrhythmia/3m	Normal	Normal	IS	c.119C > T (p.P40L),c.207+1G > T

GA, gestational age; EEG, electroencephalogram; MRI, magnetic resonance imaging; m: month; y, year; w: weeks; F, female; M, male; ES, epileptic spasms; FS, focal seizure; GTCS, generalized tonic-clonic seizures; SE, status epilepticus; MS, myoclonic seizure; ID, intellectual disability; GDD, global developmental delay; PN, pyridoxine; PLP, pyridoxal-5′-phosphate; PB, phenobarbital; VPA, valproic acid; LTG: lamotrigine; LEV, levetiracetam; PWMA, periventricular white matter attenuation; BVW, bilateral ventricles widened; CCH, corpus callosum hypoplasia; n.a, not available; IS, infantile spasms; OS, ohtahara syndrome.

a Patients 5 and 6 are twins.

### *ALDH7A1* Deficiency

In total, four patients were enrolled, including two males and two females. The phenotype analysis of this cohort identified infantile spasms in three cases (patient 1, 2, and 4) and Ohtahara syndrome in one (patient 3). Among them, two patients were born after a normal pregnancy and uneventful delivery. Patients 3 and 4 were born with hypoxia and were diagnosed with “hypoxic ischemic encephalopathy” (HIE). All patients had seizures, and the age of onset ranged from 15 days to 4 months after birth. Three patients (patient 2–4) showed ES at onset, and patient 4 also developed GTCS, focal seizures, and even status epilepticus as the disease progressed. As for patient 1, the first seizures occurred at the age of 1 month and presented as focal seizures. It was not until 7 months of age that ES occurred in clusters. Fortunately, patient 2 was given levetiracetam and pyridoxine (30 mg/day) orally the next day after onset and after 3 days of combination, the seizure was controlled. At the age of 1 year and 1 month, when she was diagnosed by genetic test, levetiracetam was gradually withdrawn and the oral pyridoxine was maintained (40 mg/day). The other three patients all underwent a tortuous process of treatment with delayed pyridoxine therapy, and seizures of two patients (patient 1 and 3) were finally controlled by pyridoxine at approximately 4 years and 1 month, respectively. Patient 4 had undergone a variety of treatments since onset and developed various forms of seizures and even status epilepticus over the course of the disease, although he presented with ES at the onset. At the age of three, he started pyridoxine treatment after genetic testing. Unfortunately, the seizure was not controlled. At the last follow up, the seizures of three patients were controlled by pyridoxine monotherapy. Patient 4 had frequent ES despite high doses of pyridoxine (300 mg/day) combined with multiple antiseizure medications (ASMs) at the age of 4 years and 2 months.

**FIGURE 2:**
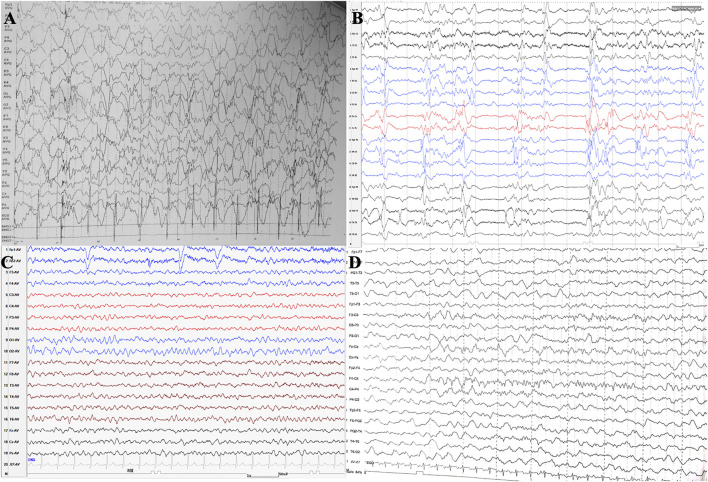
EEGs of patient 1 **(A,C)** and patient 3 **(B,D)**. **(A)** EEG showed hypsarrhythmia during frequent cluster seizures of patient 1 at the age of 2 years before pyridoxine was used. **(B)** EEG showed burst suppression of patient 3 at the age of 1 month before pyridoxine was used. **(C)** EEG of patient 1 showed normal after seizures were controlled by pyridoxine for nearly 2 years. **(D)** EEG of patient 3 showed normal after seizures were controlled by pyridoxine for nearly 2 months.

**FIGURE 3:**
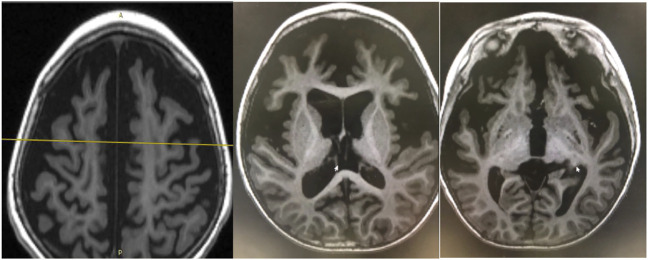
Brain MRI of patient 4. Bilateral brain atrophy due to frequent seizures and status epilepticus without pyridoxine therapy at the age of three.

EEG findings were obtained from each patient. During a period of frequent seizures, the interictal EEG of three patients indicated hypsarrhythmia (Figure 2A), and insular or series of clustered ES were captured during EEG monitoring. A suppression burst pattern on interictal EEG was indicated in patient 3 (Figure 2B). EEGs of the three patients under seizure control showed normal after pyridoxine treatment (Figures 2C,D). At the age of three, patient 4 still showed hypsarrhythmia or multifocal and generalized discharges on the EEGs after maintenance of pyridoxine for 5 months because of the frequent seizures. Brain MRI images were obtained from all patients, and the results were normal in two patients aged 2 years and 8 months and 4 months, respectively. The results of the other two cases suggested different abnormities. Brain MRI of patient 4 showed bilateral brain atrophy at the age of three (Figure 3), and patient 3 showed corpus callosum hypoplasia at 3 months of age.

At the last follow up, the age range of these four individuals was from 3 years and 5 months to 8 years and 7 months. Two patients were severe ID, and two were moderate. All patients showed global DD, including motor and language. Among them, three patients showed delayed motor development but could eventually walk independently, although with gait unsteadiness, and only one (patient 4) was still unable to reach autonomous walk at the age of 4 years and 2 months. All patients were still backward in language development. They could understand simple instructions and speak a few words occasionally, which could not satisfy the normal communication.

### *PNPO* Deficiency

We collected a cohort of 13 patients with *PNPO* mutations, nearly half of whom developed ES. There are six patients from five unrelated families, and two affected patients from two unrelated families have died. The phenotype analysis of this cohort identified infantile spasms in five cases (patients 5–6 and 8–10) and Ohtahara syndrome in one (patient 7). The current age range of these surviving four individuals was ranged from 4 years to 11 years. Four out of six patients were born prematurely (<full 37 gestational weeks). Patients 5 and 6 are twins who both experienced preterm labor and postpartum hypoxia. The age at onset of seizures was within the 24 h after birth in five patients and on the 10th day after birth in one. In four patients, the ES started on the first day of life. The other two patients presented as focal seizures and GTCS at onset and developed ES on the 40th day and the 70th day after birth, respectively. In addition to ES, all patients presented other different type of seizures: focal seizures in five patients, GTCS in three, and myoclonic seizures in two. Three patients developed status epilepticus of focal seizures.

Five patients had undergone pyridoxine treatment with or without switching to PLP, and the remaining one (patient 8) was never treated with pyridoxine or PLP and died at 1 month of age due to frequent seizures. Among the five patients receiving the initial treatment with pyridoxine, the frequency of seizures was obviously reduced in three patients and showed no significant change in two. Of the three patients who responded to pyridoxine treatment, one case (patient 10) continued to receive pyridoxine treatment in combination with other ASM, with 2–3 seizure episodes per year. At the last follow up, the dose of pyridoxine was 240 mg/day and his age was 8 years and 2 months. The other two were twins who went through the same treatment process (patients 5 and 6). Although pyridoxine combined with various ASMs could reduce the frequency of seizures, they could not be completely controlled. At the age of 6 years and 4 months, they were diagnosed with *PNPO* deficiency by gene analysis. Then PLP (30 mg/kg/day) was used instead of pyridoxine. Seizures worsened for the first few days after using PLP in both of them and returned to a similar frequency to before gradually. At the same time, due to the obvious side effects of PLP including frequent vomiting and poor appetite, pyridoxine was used again to replace PLP. When they were 8 years old, the younger twin brother (patient 2) died of frequent seizures. At the last follow up (11 years), seizures still existed in patient 1 under the treatment of pyridoxine (270 mg/day) and phenobarbital. Two patients, who had not initially responded to pyridoxine, underwent PLP trial therapy and the frequency of seizures was significantly reduced. At the last follow up, one patient maintained PLP monotherapy with a reduction in the frequency of seizures, and the other was treated with valproic acid and PLP (750 mg/day), and their seizures have been under control for more than 1 year. Overall, at the last follow up, one patient showed seizure-free after being treated by PLP combined with valproic acid, three still had infrequent seizures after being treated by PLP monotherapy or pyridoxine or PLP combined with other ASMs, and two died.

Interictal EEG records during frequent seizures were available for all patients and showed hypsarrhythmia in three, atypical hypsarrhythmia in two, and burst suppression pattern in one. After a period of treatment with pyridoxine or PLP, EEG showed either posterior discharges or atypical hypsarrhythmia at the age of 15 months in the twins. Interictal EEG of patient 7 showed slow background activity with multifocal discharges, but the age was unknown, and EEG of patient 9 showed anterior discharges at the age of 10 months. No EEG data after treatment were available in the other two patients. Brain MRI showed periventricular white matter attenuation and bilateral ventricle widening in the twins, corpus callosum hypoplasia in two others, and normal in one. The psychomotor development of four living patients was severely delayed, including severe ID and global DD.

### *PLPBP* Deficiency

**FIGURE 4:**
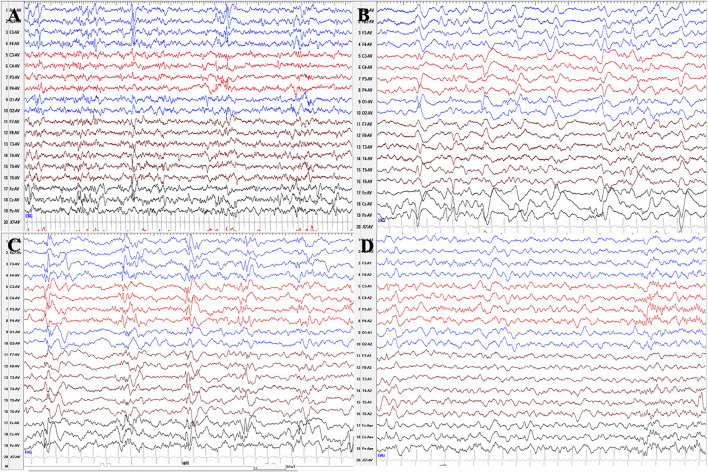
Serial EEG results of patient 11 with *PLPBP* deficiency. **(A)** and **(B)** EEGs showed multifocal sharp waves and fast waves and widely positive sharp waves in central, parietal, and midline areas at 26 days after birth before epileptic spasms occurred without pyridoxine therapy. **(C)** EEG showed multifocal and generalized spikes, spike and slow waves, and fast rhythm at the age of 3 months without pyridoxine therapy. **(D)** EEG showed normal after seizures were controlled by pyridoxine for nearly 4 months.

We collected two patients with *PLPBP* mutations, one of whom was characterized by ES. The phenotype analysis of this patient identified infantile spasms. A female aged 5 years is the second child born to nonconsanguineous Chinese parents. The patient was born with hypoglycemia, affected by her mother who suffered from gestational diabetes mellitus during pregnancy, and the blood glucose of the proband returned to normal after symptomatic treatment. Focal seizures started on the seventh day after birth and were clustered, with several episodes within 10 min. Initial EEG showed a small number of sharp waves in bilateral temporal regions. In the case of phenobarbital therapy failure, oral levetiracetam was added in combination with intravenous pyridoxine producing no seizures within 24 h. But since pyridoxine was only administered for 3 days and was not taken continuously, seizures reoccurred after pyridoxine was withdrawn for 6 days and the EEG showed multifocal sharp waves and fast waves and widely positive sharp waves in central, parietal, and midline areas at 26 days after birth (Figures 4A,B). The goal of the seizure-free patient was not achieved by adjusting the dose of levetiracetam. At 3 months after birth, re-examination of EEG showed multifocal and generalized discharges, and several episodes of ES and focal seizures were detected, even nearly close to a status epilepticus (Figure 4C). Then, topiramate was added for treatment, and pyridoxine was given intravenously at the same time. Two days later, seizures did not recur, and the same treatment was maintained for 5 days. After discharge, pyridoxine was taken orally (60 mg/day) in combination with levetiracetam and topiramate. After the genetic results were available, the ASMs were phased out and only oral pyridoxine (90 mg/day) was maintained with the patient being seizure-free. After 4 months of pyridoxine treatment, the EEG returned to normal (Figure 4D). Brain MRI showed normal. Up to the last follow up, she showed almost normal neurodevelopment at the age of five.

### Variants Information

Molecular genetic analysis revealed compound heterozygous mutations of *ALDH7A1* (NM_001182.4) in four patients, including c.1547A > G (p.Y516C) in two patients and c.1061A > G (p.Y354C), c.563T > C (p.V188A), c.1553G > C (p.R518T), c.1008+1G > A, exon 1 deletion, and exon 6 deletion in one patient, respectively. Sequence analysis of the *PNPO* (NM_018129.4) revealed a total of seven variants in six patients from five unrelated families, including c.445_448del (p.P150RfsTer27) in five patients, c.481C > T (p.R161C) in two patients, and c.748_749del (p.E251GfsTer6), c.344G > A (p.S115N), c.482G > A (p.R161H), c.233C > A (p.A78D), c.412C > T (p.R138C) in one patient, respectively. Of patient 11 who was diagnosed with *PLPBP* deficiency, two kinds of variants, c.119C > T (p.P40L) and c.207+1G > T (IVS2+1G > T), were identified (NM_007198.4). Five mutation sites of *PNPO* have not been reported. All variants of *ALDH7A1* and *PLPBP* have been reported. The parents of each patient had been available for testing and proved the autosomal recessive inheritance. The mutation was predicted to be pathogenic, based on the conservation of the amino acid and the *in silico* analysis.

## Discussion

Overall, in our 69-person cohort, the seizure presentation of 11 patients was consistent with ES, including four patients with mutations in the *ALDH7A1*, six with mutations in the *PNPO*, and one with mutation in the *PLPBP*. If classified according to syndromes, the analysis of this cohort identified nine cases presenting as infantile spasms distributed in the three mutation types, and two cases were presenting as Ohtahara syndrome with ALDH7A1 and PNPO mutations, respectively.

Infantile spasms, manifesting as ES as well as cognitive and behavioral developmental disabilities, are caused by different pathogenic events, some of which are still unknown, while others are well-recognized structural, infectious, metabolic, and immunologic defects and genetic abnormalities (Scheffer et al., 2017). In the etiologies of infantile spasms, especially genetic etiology, PDE, *PNPO* deficiency, and *PLPBP* deficiency account for only a rare proportion. Moreover, in international reports, the seizure type of ES accounted for a similar proportion of the three genetic mutations associated with vitamin B6–dependent epilepsy (Basura et al., 2009; Bennett et al., 2009; Mills et al., 2010; Scharer et al., 2010; Yeghiazaryan et al., 2012; Pérez et al., 2013; Tlili et al., 2013; Mills et al., 2014; Guerin et al., 2015; Mefford et al., 2015; van Karnebeek et al., 2016; Al Teneiji et al., 2017; Olson et al., 2017; Xue et al., 2017; Falsaperla et al., 2018; Heath et al., 2020; Jiao et al., 2020; Mohanlal et al., 2020). In our cohort, four out of the 54 patients with PDE showed ES, a rate that is less than internationally reported (Basura et al., 2009; Bennett et al., 2009; Mills et al., 2010; Scharer et al., 2010; Yeghiazaryan et al., 2012; Pérez et al., 2013; Tlili et al., 2013; Mefford et al., 2015; van Karnebeek et al., 2016; Al Teneiji et al., 2017; Falsaperla et al., 2018; Jiao et al., 2020). The number of cases carrying *PLPBP* mutations in our cohort was too small to be compared. Significantly, in our cohort of 13 patients with the *PNPO* mutation, six of them (46%) presented with ES that started early, even on the first day of life, which is significantly higher than the international norm (Mills et al., 2014; Guerin et al., 2015; Olson et al., 2017; Xue et al., 2017; Mohanlal et al., 2020). So, we considered that ES might be a common form of seizure with *PNPO* deficiency, and EEG presented as hypsarrhythmia or burst suppression pattern. In our cohort, four patients (patients 1 and 9–11) distributed in the three mutation types presented with focal seizures or GTCS at the time of onset, with ES and hypsarrhythmia EEG occurring in infancy. The patients reported by Mohanlal also showed a similar pattern to our patients, all of whom showed GTCS onset and ES occurring in infancy (Mohanlal et al., 2020). Thus, we further speculate that if seizures could be controlled early in their onset, they might not develop ES, so as to reduce the impact on development.

In addition to the onset characteristics, the treatment response of 11 patients was still related to their respective genetic mutation characteristics. According to the literatures, patients with PDE, *PLPBP* deficiency, and *PNPO* deficiency would respond to pyridoxine, PLP, or a combination of both treatments (Heath et al., 2020). It has been reported that seizure-free was reported in 87% of patients with PDE on pyridoxine monotherapy (Basura et al., 2009), but among the 31 patients with PDE previously reported by us, seizures were controlled in 97% of them (Jiao et al., 2020). In the PDE groups here, seizures were controlled by pyridoxine monotherapy in three patients, and the remaining one had refractory seizures due to secondary brain atrophy, suggesting that it is difficult for pyridoxine to control frequent seizures caused by secondary brain injury. The 79% of the patients (31/39) with *PLPBP* deficiency remained seizure‐free whether on pyridoxine or upon switching to PLP, as was observed in our cases (Heath et al., 2020). Mills et al. in their study classified patients with *PNPO* mutation into three groups, group 1 being neonatal onset seizures responding to PLP, group 2 being infantile spasms responsive to PLP, and group 3 being seizures starting under 3 months of age responding to pyridoxine (Mills et al., 2014). In their study, a patient with *PNPO* deficiency phenotypically diagnosed as infantile spasms responded well to PLP, with seizure control within 24 h but no response to pyridoxine (Mills et al., 2014). Mohanlal et al. described neonatal onset ES that respond well to PLP as opposed to pyridoxine (Mohanlal et al., 2020). In our groups with *PNPO* deficiency diagnosed with infantile spasms, patient nine underwent a conversion from pyridoxine to PLP due to pyridoxine inefficacy. Conversely, patients 5 and 6 underwent a conversion from PLP to pyridoxine, with a reduced frequency of seizures. These patients did not show an advantage in PLP treatment compared to pyridoxine. Therefore, in our *PNPO* deficiency cohort, patients with infantile spasms did not respond better to PLP than pyridoxine.

Currently, administration of adrenocorticotrophic hormone (ACTH), vigabatrin, and corticosteroids are recommended as first-line treatment for infantile spasms (Dressler et al., 2019). In Japan and European countries, pyridoxine has been recommended as the first-line treatment for infantile spasms and is widely used as the initial therapeutic agent of Ohtahara syndrome (Güveli et al., 2015). However, the use of pyridoxine in infantile spasms has always been controversial. In 2018, Kunnanayaka et al. compared the efficacy of a combination of pyridoxine and oral prednisolone versus oral prednisolone alone in 62 infants diagnosed with infantile spasms and did not find any significant difference (Kunnanayaka et al., 2018). In the literature reported by Gibaud et al., they argued that it was unreasonable to propose systematic pyridoxine or PLP trials in the classic West syndrome, since West syndrome is not a typical feature of PDE or *PNPO* deficiency (Gibaud et al., 2021). In our cohort, almost everyone presenting as infantile spasms or Ohtahara syndrome had a delay in treatment with pyridoxine or PLP. Of the PDE group, during the course of the illness, patient 4 showed ES at onset and received multiple preferred and applicable drugs for infantile spasms without becoming seizure-free. After a delay of 3 years, regular pyridoxine treatment was finally received. However, although the frequency of seizures was reduced, the seizures were not completely controlled. By contrast, in 2020, Lee et al. reported a case of PDE from Japan who showed ES and reached normal developmental milestones with oral high-dose pyridoxine treatment initiated 5 days after onset and maintained after genetic diagnosis (Lee et al., 2020). In the *PNPO* deficiency cohort of patients, patient 10, whose seizures were significantly improved with pyridoxine after a 5-year delay in treatment, the severe neurodevelopmental impact might be avoided by early treatment. Except for pyridoxine therapy, patient nine switched to PLP treatment because the seizure was unresponsive to pyridoxine, with seizure-free status maintained for more than 1 year. However, patient 8 died prematurely without treatment with pyridoxine or PLP. Therefore, although infantile spasms accounts for a relatively small number of cases in patients with vitamin B6–dependent epilepsy, the first standardized trial of pyridoxine is still necessary for such patients. Especially for patients with *PNPO* deficiency, in case of an absent response, an empirical trial of PLP could follow. In 2018, we described 11 patients diagnosed with infantile spasms who responded to pyridoxine alone, but gene testing did not indicate a genetic mutation associated with vitamin B6–dependent epilepsy (Xue et al., 2018). In 2021, Gibaud et al. also reviewed five previously reported cases of infantile spasms in which pyridoxine was effective with negative genetic results (Gibaud et al., 2021). Therefore, all patients with infantile spasms deserve to try pyridoxine therapy, not just those associated with vitamin B6-dependent epilepsy, and genetic testing could be performed if the treatment is effective. As expected, the gene results would lead to lifelong use of pyridoxine or PLP for positive ones, and withdrawal of the pyridoxine might be considered after seizure was controlled for two or more years with normal EEG for negative ones (Ohtahara et al., 2011; Xue et al., 2018).

Ohtahara syndrome often has a poor prognosis. About half of patients die in infancy, and those who survive develop infantile spasms or Lennox-Gastaut syndrome (LGS), with intractable seizures and severe neurodevelopmental delay (Beal et al., 2012). The outcome for infantile spasms also includes progression to another type of epilepsy, and transition to LGS has been reported in 18% of cases, which did not occur in our cohort (Riikonen, 1996; Riikonen, 2020). In this study, the presentation of two patients was consistent with Ohtahara syndrome. Fortunately, patient 3, diagnosed with PDE, received pyridoxine treatment more than 20 days after onset, and the seizure was controlled soon, with the EEG returning to normal. Another patient who received PLP treatment after being diagnosed with *PNPO* deficiency had a reduced frequency of seizures accompanied by an improvement in EEG at the last follow up. Olson et al. reported a case of *PNPO* deficiency with a phenotypic diagnosis of Ohtahara syndrome, whose EEG showed multifocal discharges after 1 month of treatment (the specific course of treatment is unknown) (Olson et al., 2017). Thus, it is suggested that timely and correct treatment could prevent the transition of the child from Ohtahara syndrome to subsequent epileptic encephalopathy. In the same way, early treatment of infantile spasms could also prevent the conversion to LGS or other refractory epilepsy that has not been diagnosed as epileptic syndrome.

### Limitations

Our study had several limitations. At the time of retrospective collection of medical records, the dose of intravenous pyridoxine or PLP for transient therapy and the time interval between recurrence were ambiguous in some patients before diagnosis of vitamin B6–dependent epilepsy. There is no standard dose of pyridoxine treatment for each patient. However, by describing the presentation of 11 patients consistent with ES in each mutation type, this study focuses on clinical characteristics, corresponding treatment, and prognosis, which is significant for the guidance of treatment of patients with ES.

## Conclusion

In our 69-person cohort, the seizure presentation of 11 patients was consistent with ES, including four patients with mutations in the *ALDH7A1*, six with mutations in the *PNPO*, and one with mutation in the *PLPBP*. By analyzing the presentation among them, we revealed that ES might be a common form of seizures in *PNPO* deficiency, and EEG presented as hypsarrhythmia or burst suppression pattern. In the experimental treatment of ES patients with pyridoxine, in case of an absent response, an empirical trial of PLP could follow. It is difficult for pyridoxine to control frequent seizures caused by secondary brain injury. In our *PNPO* deficiency cohort, patients with infantile spasms did not respond better to PLP than pyridoxine. Timely and correct treatment could prevent the transition of the child from Ohtahara syndrome and infantile spasms to subsequent epileptic encephalopathy or refractory epilepsy.

## Data Availability

The original contributions presented in the study are included in the article/Supplementary Material; further inquiries can be directed to the corresponding author.
